# Editorial: Current Innovations in Non-invasive Diagnoses and/or Surgical Treatments of Endometriosis

**DOI:** 10.3389/fsurg.2021.699051

**Published:** 2021-11-25

**Authors:** Jean Marc Ayoubi

**Affiliations:** Hôpital Foch, UVSQ Université Paris Saclay, Suresnes, France

**Keywords:** endometriosis, pain, AMH, MRI, metformin

## Introduction

Endometriosis is an enduring disease that causes pain and infertility. The recent years have seen several seminal new findings, which are susceptible of markedly readjusting our clinical understanding and management of the disease. The current issue of Frontiers in Surgery musters 4 important articles, which all address critical issues regarding endometriosis and its management.

## Kitajima et al.: Concentration of AMH in Peritoneal Fluid

In an imposing article, Kitajima et al. addresses striking new findings on anti-Mullerian hormone (AMH) levels in the pelvic fluid of women with endometriosis. AMH's primary function has been its long recognized role in inducing the regression of Mullerian organs – uterus and tubes – in male fetuses. More recently, the advent of high sensitivity assays revealed that AMH is produced in adult females during their reproductive years by preantral and small antral follicles. This observation led more than two decades ago to use AMH levels as a reliable quantitative marker of ovarian reserve, leading to its routine use for guiding ovarian stimulation (OS) protocols. The physiological role of AMH in adult females of reproductive age is still matter for debates however.

In a paper of this series of publications on novelties in endometriosis, Kitajima et al. propose that AMH might be involved in the pathophysiology of endometriosis. These authors propose that AMH may act as a functional mediator of female reproductive tissue derived from the Müllerian duct, such as the ovarian epithelium, pelvic peritoneum, and endometrium. Indeed, several reports describe the potential inhibitory role of AMH on normal and pathological conditions of the uterus and ovary including cancer. In their current retrospective study, Kitajima et al. report on serum and peritoneal fluid concentrations of AMH in women affected by endometriosis and disease-free controls. Their data indicate that circulating and pelvic fluid concentrations of AMH are positively correlated. Interestingly, AMH levels in PF were significantly lower in women with endometriosis as compared to findings made in control women [2.15 Å} 2.13 (mean Å} SD) vs. 4.40 Å} 4.77 ng/mL, *P* = 0.0001]. Based on their data, the authors conclude that the lower AMH levels in PF of women with advanced endometriosis suggest that AMH may be involved in the pathophysiology of peritoneal endometriosis.

## Bazot et al.: 3D MRI and Vaginal Opacification Us

The diagnosis of endometriosis – classically surgical – needs to be reassessed now that diagnostic laparoscopies are not part of routine infertility workups any more. Indeed, the sheer successes achieved by ART in the past decade has opened new *ART-first* approaches for treating infertility thereby, leading to the progressive abandon of routine diagnostic laparoscopies. The decrease in diagnostic laparoscopies has thus created a void for diagnosing endometriosis. Fortunately imaging-based approaches – MRI and ultrasounds – have made considerable progresses over the last decade to the point of now reaching replacement or near-replacement levels for the different types of endometriosis.

In the current series of articles on endometriosis, Bazot et al. address the value of 3D MRI and vaginal opacification for the diagnosis of vaginal endometriosis. This article has, amongst several interests for the readers of the journal, the advantage of assessing vaginal endometriosis following vaginal opacification by gel. In this retrospective analysis of 1,005 files, data from MRI were confronted to results of surgical exploration in a subset of 51 patients. Certain authors reported an improvement in sensitivity by the addition of contrast – vaginal gel – before conducting MRI sequences – while others challenged that concept. Yet, this improvement was only significant for junior radiologists but not for an expert (1). In their article included in this series of publications, Bazot et al., conclude that vaginal contrast increases sensitivity and specificity for diagnosis vaginal endometriosis. These findings are important for the practical management of women at risk of having vaginal endometriosis based on their suggestive symptoms.

## Maillard et al.: Systematic Review of VULVO-Peritoneal Endometriosis

In a brilliant article, Maillard et al. report a systematic review that analyses the state of the art understanding of the pathophysiology and management of vulvo-perineal endometriosis. In all, 90 studies totaling data on 283 patients are mustered in this important publication. The mean age of the patients was 32.7 years of age. Interestingly, 95.3% of these patients had a history of episiotomy or vaginal surgery. The primary symptoms experienced by women suffering from vulvo-perineal endometriosis was cyclical pain exacerbated by menstruations. Nearly all these patients underwent surgical excision of their lesions and only 10.2% presented evidence of clinical recurrence. These findings illustrate the importance of recognizing these lesions, which respond favorably to simple surgical treatment.

## Stochino-Loi et al.: Metformin for Treating Endometriosis

Endometriosis is associated with a chronic inflammation that prevails in the pelvic cavity and the eutopic endometrium residing in the uterus itself. Endometriosis is often symptomatic and existing treatment cause side effect and most importantly are all contraceptive, which prevents their use for treating endometriosis-related infertility. This therefore sparked interest for identifying new therapeutic options, which could notably be used in endometriosis patients who are trying to conceive.

Metformin has been seen as a potential candidate for treating endometriosis because of its known anti-inflammatory properties. Indeed, metformin might regulate the stroma-epithelium communication *via* Wnt2-mediated signaling in endometriosis (2). In fairly recent paper, Yilmaz et al. reported that metformin causes regression of endometriotic implants in rats (3). In their study, the activity of superoxide dismutase and tissue inhibitor of metalloproteinase-2 staining was significantly higher in metformin treated animals as compared to untreated controls. There was also a more important reductions of implant vascular endothelial growth factor levels and matrix metalloproteinase-9 in groups in treated animals (3).

In an article, which is part of the present series of publications on endometriosis, Stochino-Loi et al. report an extensive systematic review of all publications published on the effects of metformin on endometriosis. These authors notably report the only clinical trial existing, which indicates a beneficial effect of metformin treatment on the common symptoms of endometriosis such as notably, pelvic pain and dysmenorrhea (Stochino-Loi et al.).

## Author Contributions

The author confirms being the sole contributor of this work and has approved it for publication.

## Conflict of Interest

The author declares that the research was conducted in the absence of any commercial or financial relationships that could be construed as a potential conflict of interest.

## Publisher's Note

All claims expressed in this article are solely those of the authors and do not necessarily represent those of their affiliated organizations, or those of the publisher, the editors and the reviewers. Any product that may be evaluated in this article, or claim that may be made by its manufacturer, is not guaranteed or endorsed by the publisher.
